# Antifungal mechanism and transcriptome analysis of Bacillomycin D-C16 against *Fusarium oxysporum*

**DOI:** 10.3389/fmicb.2025.1698200

**Published:** 2025-11-25

**Authors:** Fuxing Lin, Yang Jiao, Hua Jiang, Yu Zheng, Shiqin Wu, Li Zhou, Xiangmei Ren, Zhaoxin Lu, Yuanhong Li

**Affiliations:** 1 School of Public Health, Xuzhou Medical University, Xuzhou, China; 2 College of Food Science and Technology, Nanjing Agricultural University, Nanjing, China

**Keywords:** Bacillomycin D-C16, *Fusarium oxysporum*, transcriptome, mitochondrial dysfunction, ROS accumulation, DNA binding

## Abstract

**Background:**

*Fusarium oxysporum* is a globally distributed soil-borne pathogen that causes substantial economic losses in cash crops. Bacillomycin D-C16, a natural antimicrobial lipopeptide produced by *Bacillus subtilis*, exhibited potent fungicidal activity against *F. oxysporum*, with a minimum inhibitory concentration (MIC) of 8 mg/L. However, the precise mechanism of its action against *F. oxysporum* remains uncharacterized.

**Methods:**

In this study, we employed transmission electron microscopy (TEM) to analyze morphological and ultrastructural alterations in *F. oxysporum* treated with Bacillomycin D-C16 and RNA-seq profiling combined with biochemical assays to elucidate Bacillomycin D-C16's mode of action against *F. oxysporum*.

**Results:**

TEM revealed that Bacillomycin D-C16 induced structural disruption of mitochondria in *F. oxysporum*. Transcriptome analysis identified 3,370 differentially expressed genes (DEGs) in *F. oxysporum*, comprising 1,488 up-regulated and 1,882 down-regulated genes. Cluster analysis revealed significant changes in gene expression patterns: DEGs associated with mitochondrial function [including oxidative phosphorylation and citrate cycle (TCA cycle) pathways] were down-regulated, while most DEGs involved in glutathione metabolism were up-regulated. Furthermore, nearly all DEGs related to DNA replication were significantly suppressed. Biochemical assays confirmed these observations: Reduced activities of mitochondrial enzymes [malate dehydrogenase (MDH), isocitrate dehydrogenase (IDH), pyruvate dehydrogenase (PDH), and complexes I–V], decreased mitochondrial membrane potential, and diminished ATP content collectively indicated mitochondrial dysfunction. Depleted glutathione (GSH) levels accompanied by elevated glutathione s-transferase (GST) activity, increased malondialdehyde (MDA) content, and accumulated reactive oxygen species (ROS) confirmed disruptions in glutathione metabolism and oxidative stress. Ultraviolet (UV) absorption spectra, fluorescence spectroscopy, and molecular docking simulations demonstrated Bacillomycin D-C16′s preferential binding to the major groove of DNA, leading to abnormal DNA replication.

**Conclusions:**

These findings collectively demonstrate that Bacillomycin D-C16 inhibits *F. oxysporum* growth through multifaceted mechanisms involving transcriptional regulation, mitochondrial impairment, ROS accumulation, and interference with DNA replication.

## 1 Introduction

*Fusarium oxysporum*, a common soil-borne fungal, is recognized as one of the most widely spread plant pathogens among the *Fusarium* genus (Zhang D. et al., 2024). *F. oxysporum* can infect the roots, stems, leaves, flowers, and fruits of crops, and cause substantial economic losses in many economically important crops including rice, wheat, cotton, cucumbers, tomatoes, etc. (Han et al., 2019; Li et al., 2023). Till date, chemical synthetic fungicides including carbendazim, hexaconazole, diniconazole, and prothioconazole could effectively control *F. oxysporum* (Hudson et al., 2021; Sharma et al., 2021). However, the excessive use of synthetic fungicides poses risks to human health and the environment, and can even lead to the emergence of fungicide-resistant strains (Hudson et al., 2021).

Promising results have been achieved using *Bacillus* species as antagonists to control *F. oxysporum*, owing to their production of natural antifungal peptides (Boulahouat et al., 2023). For instance, Fengycin B, produced by *Bacillus subtilis* FAJT-4, exhibited antifungal activity against *F. oxysporum*, with a minimum inhibitory concentration (MIC) of 0.25 mg/mL (Deng et al., 2024). Bacillomycin D, a cyclic lipopeptide produced by *Bacillus subtilis*, is characterized asa heptapeptide core covalently linked to a β-amino fatty acid chain (14–17 carbons) that confers broad-spectrum antifungal activity (Jin et al., 2020; Qian et al., 2016, 2025). Unlike conventional synthetic fungicides, Bacillomycin D avoids the adverse environmental impacts of chemical agents and shows low propensity for resistance development due to its membrane-targeting mechanism (Lin et al., 2022). Bacillomycin D presents promising prospects for the advancement of an antifungal agent.

Previous studies have demonstrated that the antifungal action of Bacillomycin D primarily targets fungal cell membrane integrity (Lin et al., 2022; Liu et al., 2025; Wu et al., 2020). Specifically, Bacillomycin D destabilizes the lipid bilayer by binding to ergosterol, inducing dysregulation of membrane permeability, fluidity, and structural integrity, which triggers cellular content leakage and irreversible cell death (Wu et al., 2020). Additionally, Gu et al. (2017) proposed a complementary mechanism involving ROS overproduction, deoxynivalenol accumulation, and activation of mitogen-activated protein kinase signaling pathways. Building on this, our previous research has established that Bacillomycin D-C16 (a Bacillomycin D monomer) induces cell death in *Fusarium verticillioides* by destroying cell membranes and promoting ROS accumulation (Lin et al., 2022). However, few studies have investigated the molecular interactions between Bacillomycin D monomers and *F. oxysporum*. Notably, our preliminary data showed that the minimum inhibitory concentrations (MICs) of Bacillomycin D-C16, -C15, and -C14 against *F. oxysporum* were 8 mg/L, 16 mg/L, and 32 mg/L, respectively (Supplementary Figure S1A). This indicates that the inhibitory effect of Bacillomycin D is positively correlated with the length of its fatty acid chain. Despite its proven efficacy, the precise mechanism of action against *F. oxysporum* remains uncharacterized.

High-throughput RNA sequencing (RNA-seq) enables the identification of genes and regulatory networks modulated by antifungal peptides, providing insights into the molecular mechanisms underlying their effects on fungal cells (Fan et al., 2024). The lipopeptide mycosubtilin-C17 damages *Verticillium dahliae* cell membranes and walls while inducing mitochondrial swelling; these disruptions occur by regulating differentially expressed genes associated with membrane/wall synthesis, cell cycle progression, and energy metabolic pathways, ultimately inhibiting fungal growth (Zhang et al., 2023). Via RNA-seq analysis, Jin et al. (2024) demonstrated that a crude lipopeptide extract from *Bacillus velezensis* TCS001 confers antifungal activity through suppression of *Bcpsd* expression in *Botrytis cinerea*. In this study, we employed transmission electron microscopy (TEM) to analyze morphological and ultrastructural alterations in *F. oxysporum* treated with Bacillomycin D-C16 and RNA-seq profiling combined with biochemical assays to elucidate Bacillomycin D-C16′s mode of action against *F. oxysporum*. This study not only enriched the antifungal mechanism theory of Bacillomycin D-C16 but also established a scientific foundation for its potential applications in disease control.

## 2 Materials and methods

### 2.1 Fungus

*F. oxysporum* (ACCC 38875) was obtained from the Agricultural Culture Collection of China (ACCC, China). It was cultured in potato dextrose broth (PDB) at 25 °C. Mature spores were then generated on potato dextrose agar (PDA), suspended in sterile distilled water, and counted using a hemocytometer.

### 2.2 Bacillomycin D-C16

*Bacillus subtilis* fmbJ (CGMCC 0943) was cultured in YIGL medium (4.52 g yeast extract/L, 42.62 g inulin/L, 5 g L-glutamine/L, 7 g calcium L-lactate/L) at 33°C and 180 rpm for 120 h. After centrifugation, fermentation broth was treated with 6 M HCl to precipitate. The precipitate was redissolved in methanol and centrifuged to obtain crude extract. Bacillomycin D-C16 was purified by preparative HPLC using an XBridge™ Prep C18 column. The mobile phase consisted of (A) water and (B) acetonitrile. A 1 mL sample was loaded and eluted with a linear gradient: 30%−45% B over 15 min, followed by 45%−55% B over 35 min, at a flow rate of 4 mL/min. Elution was monitored by UV detection at 225 nm (Lin et al., 2019).

### 2.3 Transmission electron microscope analysis

*F. oxysporum* spore suspension (1 × 10^6^ spores/mL) was inoculated into PDB and cultured at 25 °C. The culture was then filtered to harvest the mycelia. A total of 500 mg of mycelia were resuspended in phosphate-buffered saline (PBS) and treated with Bacillomycin D-C16 at a final concentration of 0 mg/L (control) or 8 mg/L for 24 h at 25 °C. After treatment, the mycelia were fixed in 2.5% (v/v) glutaraldehyde solution and prepared for ultrastructural observation using TEM (Tecnai G2 Spirit Twin, FEI, USA).

### 2.4 RNA-seq of *F. oxysporum*

*F. oxysporum* mycelia were exposed to Bacillomycin D-C16 at a final concentration of 0 or 8 mg/L and incubated at 25 °C for 12 h. After washing three times with 10 mmol/L PBS, the mycelia were collected, flash-frozen in liquid nitrogen, and stored at −80 °C for subsequent RNA-seq experiments.

#### 2.4.1 RNA-seq transcriptome library construction

Total RNA in *F. oxysporum* mycelia was extracted using the MJZol total RNA extraction kit (Majorbio, Shanghai, China). RNA quality was assessed using a 5300 Bioanalyzer, and concentration was quantified with the NanoDrop 2000. The RNA-seq transcriptome library was prepared following Illumina Stranded mRNA Prep (Illumina, San Diego, USA) using 1 μg of total RNA. Shortly, mRNA was isolated via polyA selection [oligo(dT) beads], then fragmented with fragmentation buffer. Double-stranded cDNA was synthesized using random hexamer primers, followed by end-repair, phosphorylation and adapter addition per library construction protocol. Libraries were size-selected (300–400 bp, magnetic beads) and PCR-amplified (10–15 cycles). After quantification with Qubit 4.0, the library was sequenced on NovaSeq X Plus (PE150) using NovaSeq Reagent Kit.

#### 2.4.2 RNA-seq data analysis

To identify differential expression genes (DEGs) between Bacillomycin D-C16 treatment and control, the expression level of each transcript was calculated according to the transcripts per million reads (TPM) method. DEGs with |log2 fold change (FC)| ≧ 1 and P-adjust < 0.05 were considered to be significantly different expressed genes. GO functional enrichment and KEGG pathway analysis conducted using Goatools (version 0.6.5) and KOBAS (version 3.0) (Xie et al., 2011).

#### 2.4.3 qRT-PCR verification

To validate the reliability of the RNA-Seq transcript-level results, seven unigenes were randomly selected for qRT-PCR verification. *F. oxysporum* mycelia were prepared using a method similar to that employed in the RNA-Seq experiments. Total RNA was extracted using the RNA isolation kit and reverse-transcribed into cDNA. qRT-PCR was performed on the StepOne Plus System (7500, Applied Biosystems, USA) using the SYBR Green Master Mix. Relative gene expression levels were determined using the 2^−ΔΔ*C*(*T*)^ method (Livak and Schmittgen, 2001). The elongation factor 1-α (*EF1-*α) acted as the internal control (Zhang et al., 2018). qRT-PCR primer pairs are listed in Supplementary Table S1, and all experiments were conducted with three biological replicates.

### 2.5 ATP content and mitochondrial membrane potential assays

ATP content in *F. oxysporum* mycelia treated with Bacillomycin D-C16 (0, 4, 8, and 16 mg/L) was quantified using an ATP assay kit (Beyotime Biotechnology, Shanghai, China).

The effect of Bacillomycin D-C16 on the MMP of *F. oxysporum* mycelia was detected by the probe Rhodamine 123 (Zhao et al., 2022). Mycelia were treated with 0 mg/L (control) or 8 mg/L Bacillomycin D-C16, incubated with 2 μmol/L Rhodamine 123 solution for 30 min, washed three times with PBS, and imaged under a fluorescence microscope (Olympus IX83, Tokyo, Japan).

### 2.6 Mitochondrial enzymes activities determination

Enzyme activity assays of NADH CoQ reductase (complex I), succinate-coenzyme Q reductase (complex II), CoQ-cytochrome C reductase (complex III), cytochrome C oxidase (complex IV), ATP synthase (complexes V), malate dehydrogenase (MDH), isocitrate dehydrogenase (IDH), and pyruvate dehydrogenase (PDH) of *F. oxysporum* mycelia treated with 0, 4, 8, and 16 mg/L Bacillomycin D-C16 were determined according to Yun et al. (2023) using the Assay Kit (Solarbio, Beijing, China).

### 2.7 Malondialdehyde (MDA) content and reactive oxygen species (ROS) determination

The mycelia (500 mg) were treated with 0, 4, 8, and 16 mg/L Bacillomycin D-C16 and incubated at 25°C for 12 h. MDA content was measured following the manufacturer's instructions (MDA Assay Kit, Solarbio, Beijing, China).

To determine whether ROS accumulated in *F. oxysporum* mycelia after expose to Bacillomycin D-C16, we employed the fluorescent probe dichlorodihydrofluorescein diacetate (DCFH-DA) combined with fluorescence microscopy. *F. oxysporum* mycelia were treated with 0 or 8 mg/L Bacillomycin D-C16 and subsequently incubated with 10 μM DCFH-DA for 30 min. The mycelia were observed using an Olympus IX83 fluorescence microscope (Tokyo, Japan).

### 2.8 Glutathione content and glutathione s-transferase activity determination

*F. oxysporum* mycelia were treated with Bacillomycin D-C16 at concentrations of 0, 4, 8, and 16 mg/L. After a 12 h incubation, the mycelia were collected by filtration. The GST activity and GSH content in *F. oxysporum* were measured using a Versa Max spectrophotometer (Molecular Devices, USA), following the manufacturer's instructions provided by Solarbio (Beijing, China) (OuYang et al., 2018). Each treatment was performed in three independent replicates.

### 2.9 Effects of ROS scavengers on the activity of Bacillomycin D-C16

A 50 μL suspension of *F. oxysporum* spores (2 × 106 spores/mL) was dispensed into each well of a 96-well plate. Bacillomycin D-C16 was serially diluted in PDB to achieve final concentrations ranging from 0 to 256 mg/L (0, 1, 2, 4, 8, 16, 32, 64, 128, and 256 mg/L). Four experimental groups were established: a Bacillomycin D-C16-only control group, and three treatment groups supplemented with 5 mmol/L vitamin C (VC), GSH, or N-acetyl-cysteine (NAC). The MIC for each group was defined as the lowest drug concentration that inhibited visible microbial growth (Lin et al., 2022).

### 2.10 Ultraviolet spectroscopy determination

The interaction between Bacillomycin D-C16 and *F. oxysporum* mycelia genomic DNA was assessed using a modified method adapted from Liu et al. (2019a). Absorption spectral titrations were conducted by maintaining a constant Bacillomycin D-C16 concentration (4 mg/L) in 0.01 mol/L PBS, while DNA concentration (0, 1.25, 2.5, 5, 10, and 20 ng/μL) was progressively increased. Samples were equilibrated at 25 °C for 10 min. Spectral measurements were recorded within the 200–320 nm wavelength range using a SPARK^®^ Tecan spectrophotometer (Switzerland).

### 2.11 Fluorescence spectroscopy assays

Bacillomycin D-C16 was kept constant at 4 mg/L in 0.01 mol/L PBS, while the DNA concentration (0, 1.25, 2.5, 5, 10, and 20 ng/μL) was gradually increased (Liu et al., 2019a). After incubation at 25 °C for 30 min, the emission spectra were assessed over the 300–450 nm range using a SPARK^®^ Tecan spectrophotometer (Switzerland).

### 2.12 Molecular docking of Bacillomycin D and DNA

The Bacillomycin D-C16 structure was obtained by ChemDraw 19.0, and B-DNA (CGCGAATTCGCG)_2_ was retrieved from the Protein Data Bank (PDB ID: 1BNA). Prior to docking, both molecules were preprocessed with AutoDock Vina 1.2.2, which included hydrogen addition and charge assignment. A Lamarckian genetic algorithm was employed as the stochastic search engine for molecular docking (Liu et al., 2019a). The conformation with the lowest binding energy was selected as the optimal binding mode and further visualized using PyMOL.

### 2.13 Statistical analysis

Statistical analyses were conducted using Tukey's Honest Significant Difference (HSD) test in IBM SPSS Statistics 27 (Midway et al., 2020). *P* < 0.05 indicated statistically significant difference.

## 3 Results

### 3.1 Effect of Bacillomycin D-C16 on the morphology and structure of *F. oxysporum*

**Figure 1:**
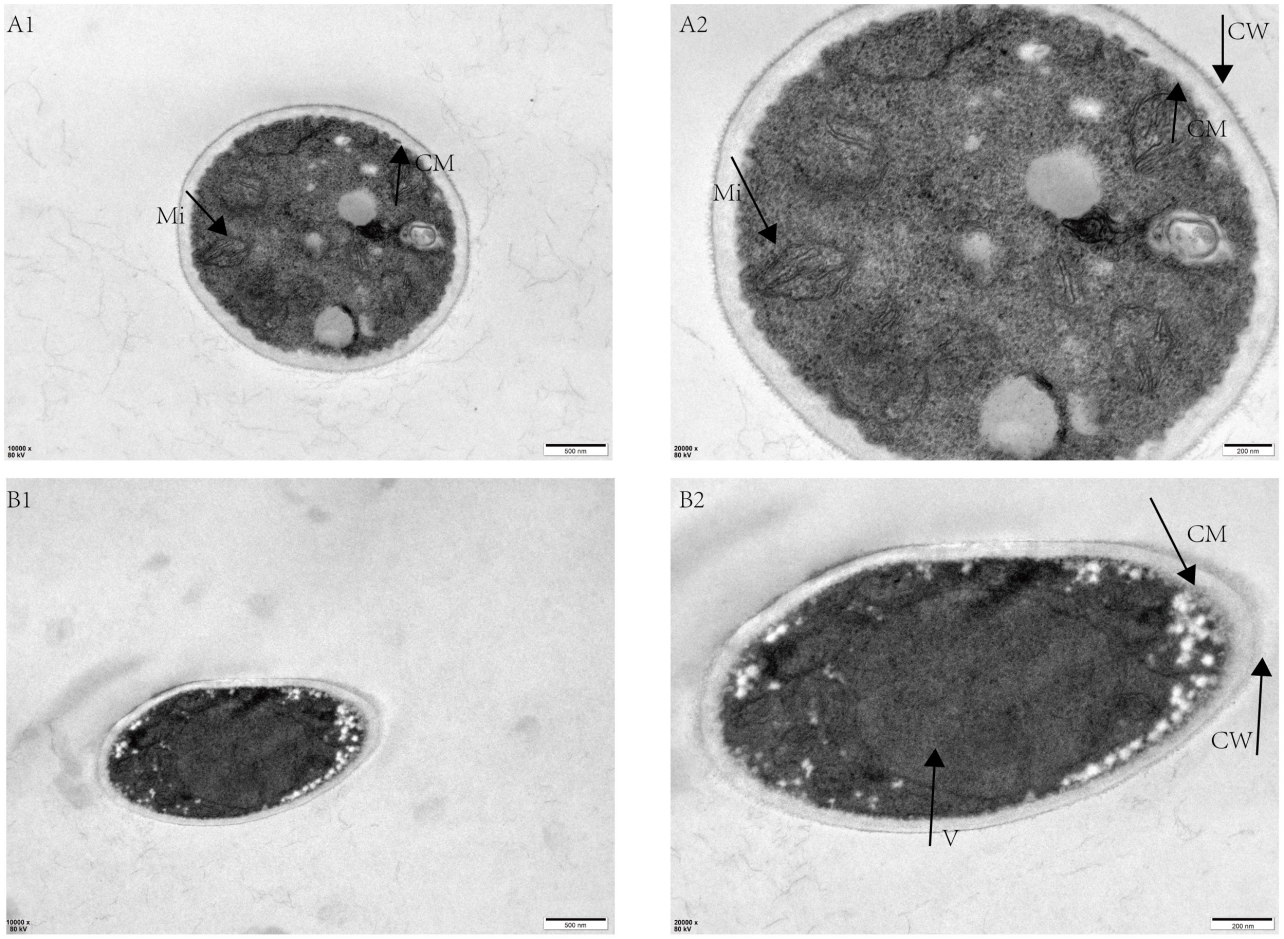
Transmission electron microscope images of *F. oxysporum* mycelia treated with varying concentrations of Bacillomycin D-C16. **(A1, A2)**, control group (no Bacillomycin D-C16); **(B1, B2)**, treatment group (8 mg/L Bacillomycin D-C16). Mi, mitochondria; CM, cell membrane; V, vacuole; CW, cell wall.

The morphological and structural effects of Bacillomycin D-C16 on *F. oxysporum* mycelia were analyzed using TEM (Figure 1). Untreated *F. oxysporum* mycelia exhibited intact cellular architecture, with abundant organelles uniformly distributed in the cytoplasm, well-preserved cell membranes, structurally intact cell walls, and clearly defined mitochondrial structures (Figures 1A1, A2). However, treatment with 8 mg/L Bacillomycin D-C16 induced significant ultrastructural alterations. The cell wall and membrane were dissolved, becoming fuzzy, and were accompanied by a marked reduction or dissolution of cytoplasmic organelles, and extensive vacuolization within the cells. Notably, mitochondrial structures were completely disrupted (Figures 1B1, B2).

### 3.2 RNA-seq analysis of *F. oxysporum*

#### 3.2.1 Quality assessment of transcriptome data

The transcriptome sequencing dataset from Bacillomycin D-C16-treated group and the control group showed a total of 36.42 Gb of clean data (Supplementary Table S2). The raw and clean reads in each sample ranged from 41,026,826 to 51,950,532 and 40,618,524 to 51,496,994, respectively. The Q20% and Q30% values exceeded 97.77% and 93.38%, respectively. The GC content averaged 52.14%.

#### 3.2.2 Overview of differentially expressed genes

**Figure 2:**
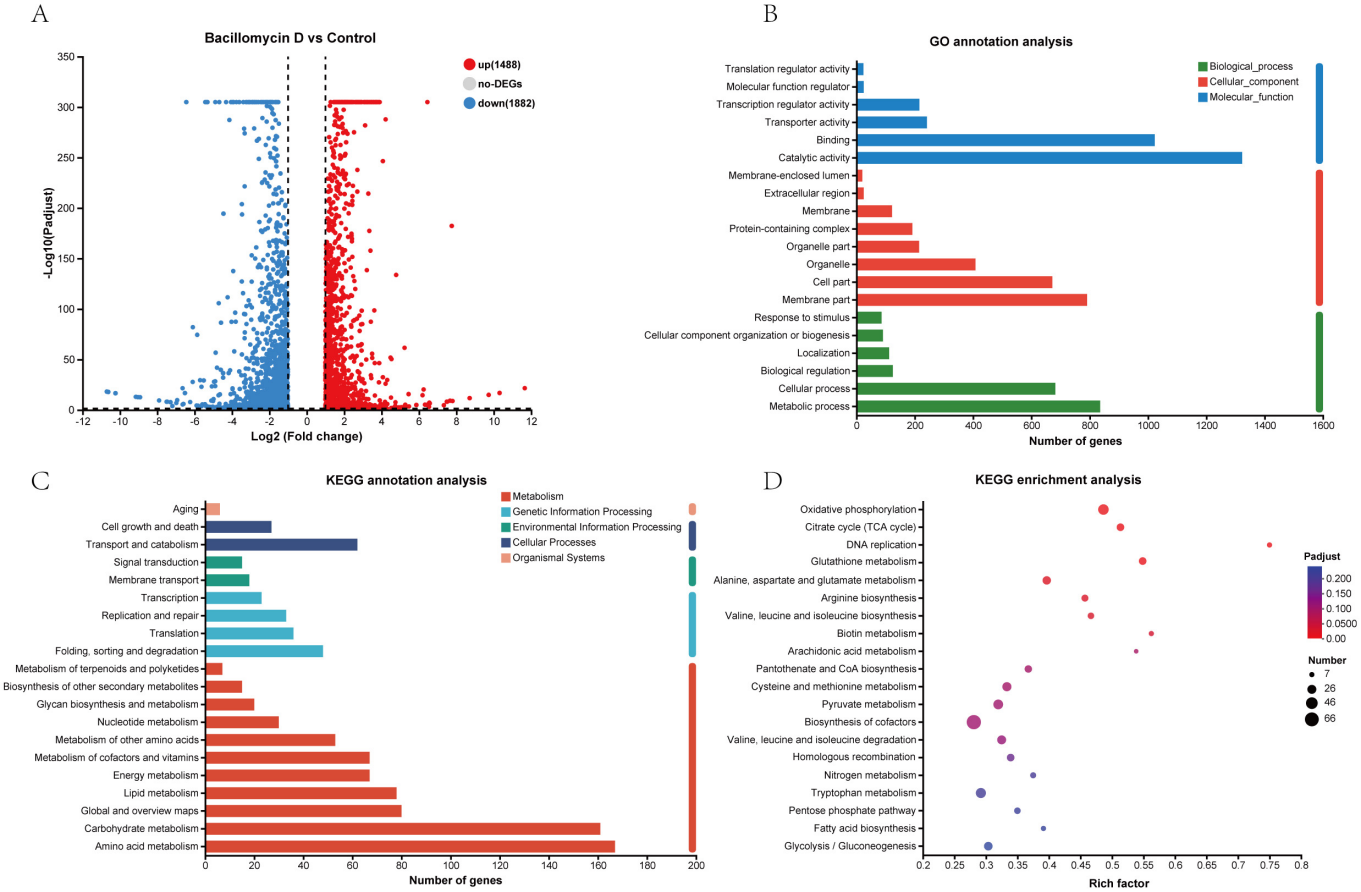
Analysis of differentially expressed genes (DEGs). **(A)** Volcano plot analysis identifies 1,488 up-regulated and 1,882 down-regulated genes. **(B)** Gene ontology (GO) functional enrichment analysis of DEGs. **(C)** Kyoto encyclopedia of genes and genomes (KEGG) pathway annotation. **(D)** KEGG enrichment analysis of significantly altered pathways.

DEGs were defined using two criteria: a twofold difference in transcript levels between treated and control mycelia and a P-adjusted value ≤ 0.05 (Supplementary Table S3). From the results, 3,370 DEGs were identified in the Bacillomycin D-C16 treatment group compared to the control. These comprised 1,488 significantly upregulated genes and 1,882 significantly downregulated genes (Figure 2A).

#### 3.2.3 Functional analysis of the DEGs

The results of Gene Ontology (GO) annotation, Kyoto Encyclopedia of Genes and Genomes (KEGG) annotation, and KEGG enrichment analysis for DEGs were presented in Figure 2. GO annotation analysis revealed that the primary biological processes associated with DEGs included metabolic processes and cellular processes, while their main cellular components were mostly distributed in membrane part and cell part. The predominant molecular functions were catalytic activities and binding (Figure 2B). KEGG annotation analysis further revealed that DEGs were predominantly involved in transport and catabolism, membrane transport, protein folding/sorting/degradation, amino acid metabolism, and carbohydrate metabolism (Figure 2C). KEGG enrichment analysis demonstrated significant enrichment of DEGs in oxidative phosphorylation, citrate cycle (TCA cycle), DNA replication, and glutathione metabolism pathway (Figure 2D).

#### 3.2.4 Evaluation of DEGs related to mitochondrial function

**Figure 3:**
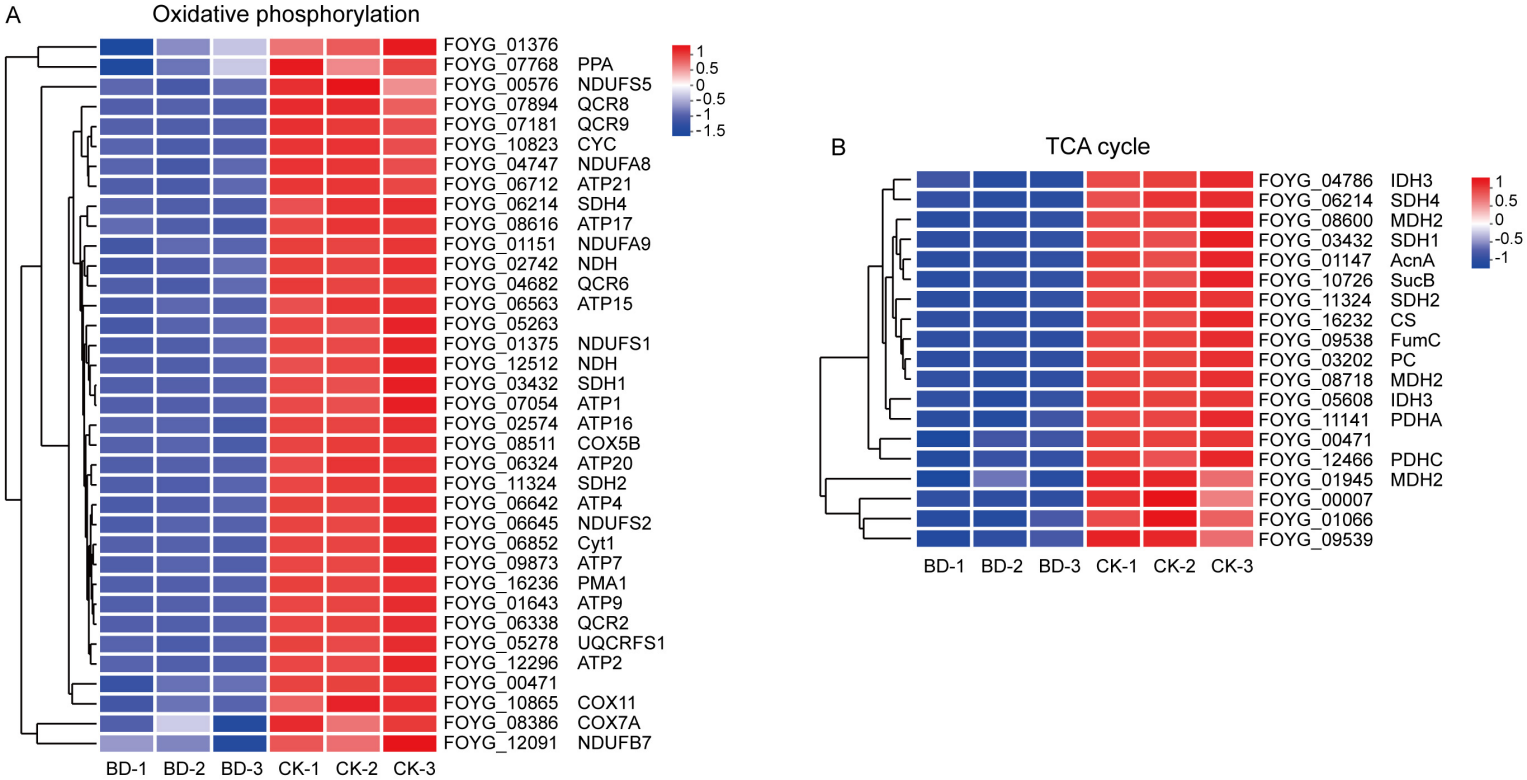
Heatmaps illustrating relative expression levels of DEGs associated with oxidative phosphorylation **(A)** and the TCA cycle **(B)**. Blue: down-regulated; red: up-regulated. Each row corresponds to an individual DEG, while columns represent biological replicate samples. BD, Bacillomycin D-C16; CK, control.

Bacillomycin D-C16 altered the expression of genes associated with mitochondrial function (Figure 3, Supplementary Tables S4, S5). Thirty-six DEGs involved in oxidative phosphorylation were significantly down-regulated (2.03- to 32.25-fold). These included 9 DEGs encoding subunits of Complex I (NDUFB7, NDUFA8, NDUFA9, NDUFS1, NDUFS2, NDUFS5, and NDH), 3 DEGs encoding subunits of Complex II (SDH1, SDH2, and SDH4), 7 DEGs encoding subunits of Complex III (QCR2, QCR6, QCR8, QCR9, Cyt1, CYC, and UQCRFS1), 3 DEGs encoding subunits of Complex IV (COX5B, COX7A, and COX11), and 12 DEGs encoding subunits of Complex V (ATP1, ATP2, ATP4, ATP7, ATP9, ATP15, ATP16, ATP17, ATP20, ATP21, and PMA1). Additionally, 1 DEG encoding inorganic pyrophosphatase (PPA) was down-regulated.

All genes enriched in the TCA cycle pathway exhibited significant down-regulated (2.09- to 32.25-fold), including 3 DEGs encoding malate dehydrogenase (MDH), 3 DEGs encoding succinate dehydrogenase (SDH), 1 DEG encoding citrate synthase (CS), 2 DEGs encoding isocitrate dehydrogenase (IDH), 1 DEG encoding aconitase (ACN), 1 DEG encoding succinyl-CoA synthetase (SUC), 1 DEG encoding fumarase (FUM), 1 DEG encoding pyruvate carboxylase (PC), and 2 DEGs encoding pyruvate dehydrogenase (PDH).

Collectively, these findings suggest that Bacillomycin D-C16 may suppress the transcriptional activity of key genes, thereby disrupting oxidative phosphorylation and the TCA cycle in *F. oxysporum*.

#### 3.2.5 Evaluation of DEGs associated with glutathione metabolism

**Figure 4:**
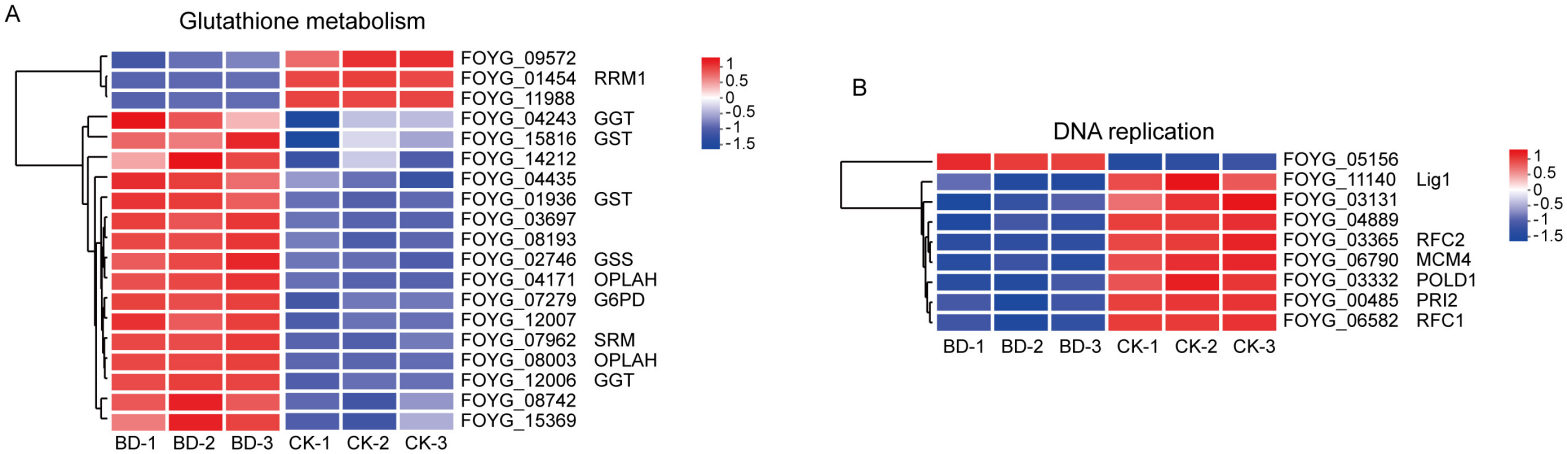
Heatmaps illustrating relative expression levels of DEGs related to glutathione metabolism **(A)** and DNA replication **(B)**. Blue: down-regulated; red: up-regulated. Each row corresponds to an individual DEG, while columns represent biological replicate samples. BD, Bacillomycin D-C16; CK, control.

As shown in Figure 4A and Supplementary Table S6, 19 DEGs were enriched in the glutathione metabolism pathway, with 16 genes showing up-regulation (2.08- to 13.89-fold) and 3 genes exhibiting down-regulation (4.53- to 6.67-fold). These findings suggest that Bacillomycin D-C16 may activate the glutathione metabolism pathway through transcriptional regulation of associated genes.

#### 3.2.6 Evaluation of DEGs involved in DNA replication

Bacillomycin D-C16 altered the expression levels of specific genes associated with DNA replication (Figure 4B and Supplementary Table S7). RNA-seq analysis identified 9 differentially expressed genes (DEGs) annotated to DNA replication in Bacillomycin D-C16-treated cells. Among these, 8 DEGs were significantly down-regulated, including FOYG_03131, FOYG_04889, FOYG_03365, FOYG_06790, FOYG_03332, FOYG_00485, FOYG_06582, and FOYG_11140. These results suggest that Bacillomycin D-C16 may inhibit DNA replication in *F. oxysporum*, thereby suppressing normal fungal growth.

#### 3.2.7 qRT-PCR verification

To validate the reliability of RNA-seq expression levels, 7 DEGs were selected for qRT-PCR verification. The selected DEGs comprised *NDH* (FOYG_02742), *ATPase* (FOYG_16236), *Cyt1* (FOYG_06852), *CYC* (FOYG_10823), *MDH* (FOYG_08718), *IDH* (FOYG_05608), and *GST* (FOYG_01936). The qRT-PCR results demonstrated strong concordance with RNA-seq data (Supplementary Figure S2), confirming the accuracy of the transcriptomic data.

### 3.3 Effects of Bacillomycin D-C16 on mitochondrial function

**Figure 5:**
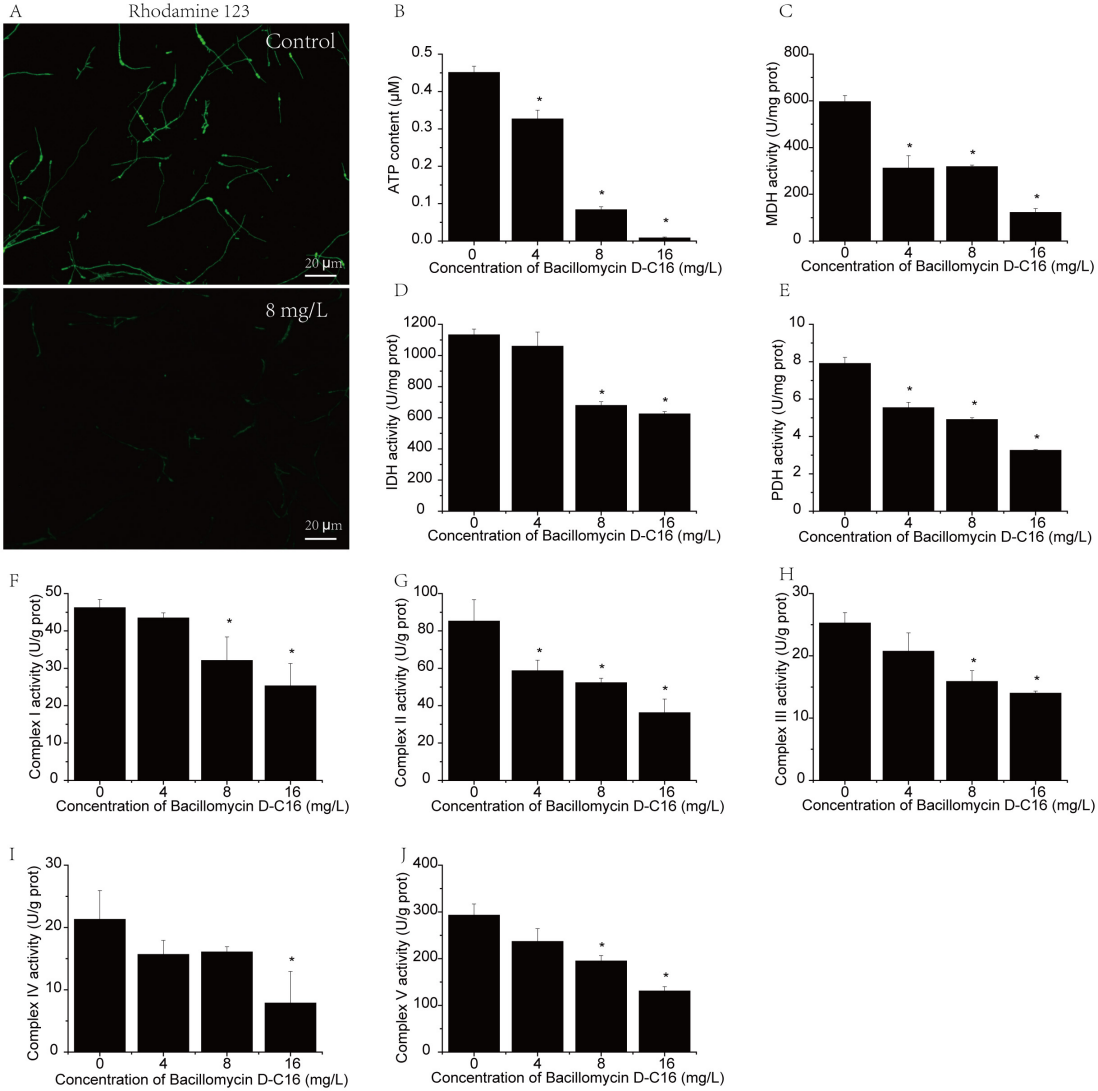
Effects of Bacillomycin D-C16 on mitochondrial function in *F. oxysporum*. Mitochondrial membrane potential (MMP) visualized in mycelia using Rhodamine 123 staining **(A)**. Quantification of ATP content **(B)**, enzymatic activities of malate dehydrogenase [MDH, **(C)**], isocitrate dehydrogenase [IDH, **(D)**], pyruvate dehydrogenase [PDH, **(E)**], and mitochondrial electron transport chain complexes I–V **(F–J)** in *F. oxysporum* after Bacillomycin D-C16 treatment. Data are expressed as mean ± SD (*n* = 3); error bars represent standard deviations. Asterisks (*) denote significant differences compared to the untreated control (*P* < 0.05).

As shown in Figure 5A, the fluorescence intensity of *F. oxysporum* mycelia showed significant reduction (*P* < 0.05) following treatment with 8 mg/L Bacillomycin D-C16. The results demonstrated Bacillomycin D-C6′s capacity to reduce mitochondrial membrane potential (MMP) in fungal cells, directly impairing mitochondrial functionality.

Mitochondria serve as central hubs for energy production and metabolic regulation in eukaryotic cells, with oxidative phosphorylation being their core function for ATP synthesis. The mitochondrial electron transport chain, composed of Complexes I–V, generates approximately 95% of cellular ATP. Following treatment with Bacillomycin D-C16, the enzymatic activities of Complexes I–V exhibited significant reductions compared to untreated controls (Figures 5F–J). At a concentration of 16 mg/L Bacillomycin D-C16, the activities of Complexes I–V decreased to 25.30, 36.24, 14.00, 7.86, and 130.85 U/g prot, respectively, representing reductions of 45.27% (Complex I), 57.49% (Complex II), 44.58% (Complex III), 63.13% (Complex IV), and 55.3% (Complex V) relative to control values (46.23, 85.25, 25.26, 21.32, and 292.91 U/g prot, respectively). These pronounced declines in electron transport chain activity directly correlated with diminished ATP production (Figure 5B), underscoring Bacillomycin D-C16′s disruptive effects on mitochondrial bioenergetics.

The enzymatic activities of MDH, IDH, and PDH in TCA cycle were illustrated in Figures 5C–E. In *F. oxysporum* treated with Bacillomycin D-C16, the activities of MDH, IDH, and PDH demonstrated a concentration-dependent decline. At 8 mg/L Bacillomycin D-C16, the activities decreased significantly to 318.45 U/mg prot (MDH), 678.79 U/mg prot (IDH), and 4.90 U/mg prot (PDH), representing reductions of 46.60%, 40.09%, and 38.05%, respectively, compared to untreated controls (596.37 U/mg prot, 1,132.93 U/mg prot, and 7.91 U/mg prot). Maximum inhibition occurred at 16 mg/L Bacillomycin D-C16, with MDH, IDH, and PDH activities plummeting to 122.05 U/mg prot, 625.12 U/mg prot, and 3.25 U/mg prot, respectively. These values correspond to dramatic reductions of 79.53% (MDH), 44.82% (IDH), and 58.91% (PDH) relative to control levels, confirming severe disruption of TCA cycle functionality.

The above results demonstrat that Bacillomycin D-C16 induced mitochondrial dysfunction by collapsing the transmembrane electrochemical gradient, subsequently inhibiting electron transport chain activity in the respiratory pathway. This dual disruption of membrane potential and oxidative phosphorylation ultimately led to energy metabolism failure, resulting in fungal cell death.

### 3.4 Effect of Bacillomycin D-C16 on oxidative metabolism

**Figure 6:**
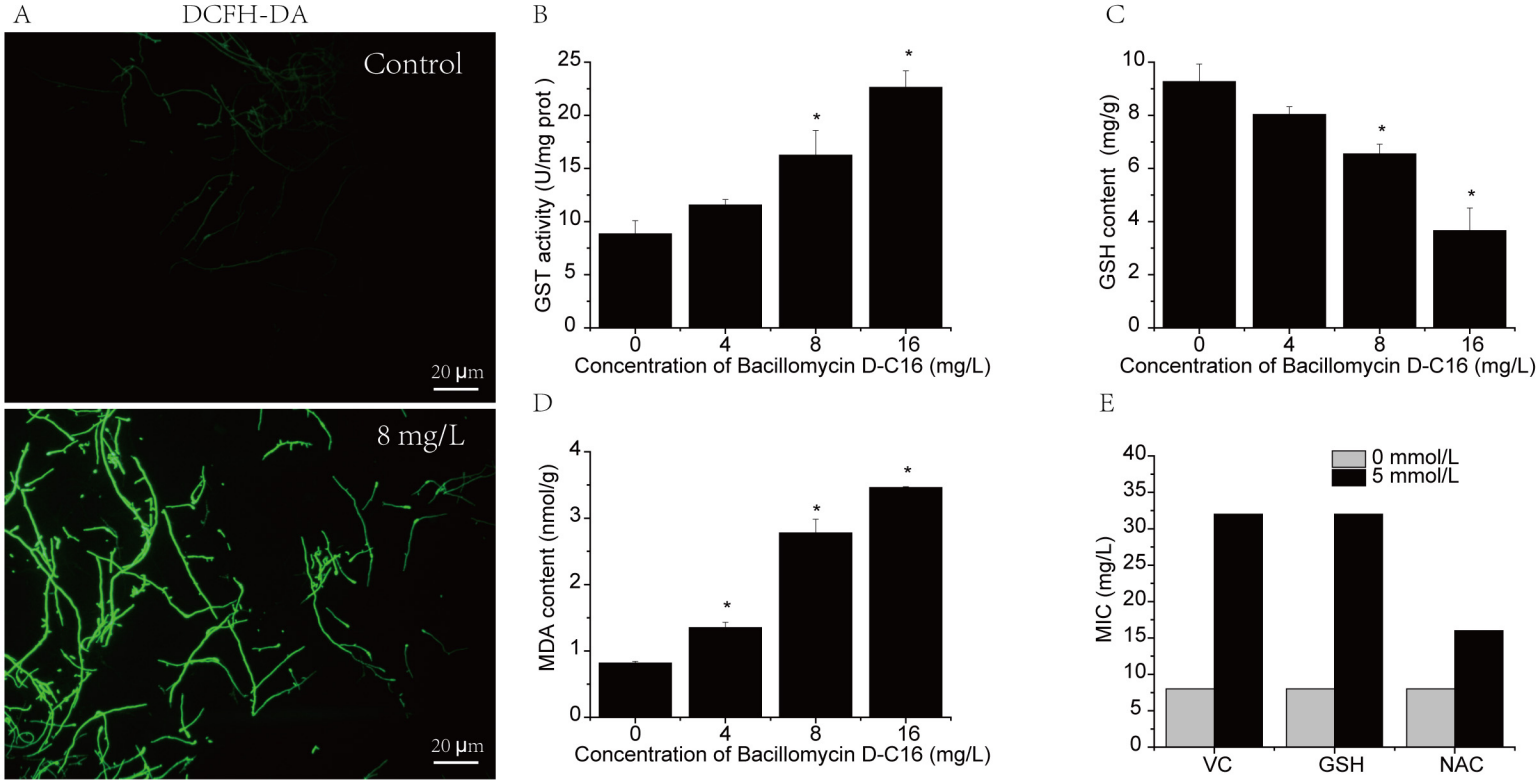
Effect of Bacillomycin D-C16 on oxidative metabolism in *F. oxysporum*. Reactive oxygen species (ROS) production in *F. oxysporum* mycelia treated with the fluorescent probe DCFH-DA **(A)**. Effects of Bacillomycin D-C16 on glutathione S-transferase (GST) activity **(B)**, reduced glutathione (GSH) content **(C)**, and malondialdehyde (MDA) accumulation **(D)** in *F. oxysporum*. Influence of ROS scavengers vitamin C (VC), GSH, and N-acetylcysteine (NAC) on the minimum inhibitory concentration (MIC) of Bacillomycin D-C16 against *F. oxysporum* **(E)**. Asterisks (*) indicate significant differences (*P* < 0.05) compared to the control. Experiments were performed in triplicate.

As shown in Figure 6B, the GST activity in *F. oxysporum* mycelia increased significantly with rising Bacillomycin D-C16 concentrations compared to untreated control. In contrast, the GSH content in the mycelium decreased significantly as the Bacillomycin D-C16 concentration increased (Figure 6C).

The accumulation of ROS in *F. oxysporum* mycelia following Bacillomycin D-C16 treatment was visualized using the fluorescent probe DCFH-DA (Figure 6A). Untreated mycelium exhibited negligible fluorescence intensity, indicative of basal ROS levels. In contrast, treatment with 8 mg/L Bacillomycin D-C16 induced a pronounced increase in fluorescence intensity, reflecting ROS overproduction.

MDA content, a biomarker of lipid peroxidation, exhibited a concentration-dependent elevation under Bacillomycin D-C16 exposure (Figure 6D). At concentrations of 4, 8, and 16 mg/L, MDA levels in treated mycelia were significantly higher than those in the untreated group (*P* < 0.05), confirming oxidative membrane damage proportional to antifungal agent dosage.

To determine whether the antifungal activity of Bacillomycin D-C16 against *F. oxysporum* is mediated by intracellular ROS accumulation, we employed three ROS scavengers: VC, GSH, and NAC. Treatment with 5 mmol/L VC, GSH, or NAC increased the MIC of Bacillomycin D-C16 by 4-fold, 4-fold, and 2-fold, respectively (Figure 6E). The elevated Bacillomycin D-C16 concentrations required for fungal inhibition in the presence of ROS scavengers suggest a positive correlation between its antifungal activity and intracellular ROS levels.

### 3.5 Effect of Bacillomycin D-C16 on DNA

#### 3.5.1 UV spectroscopy

**Figure 7:**
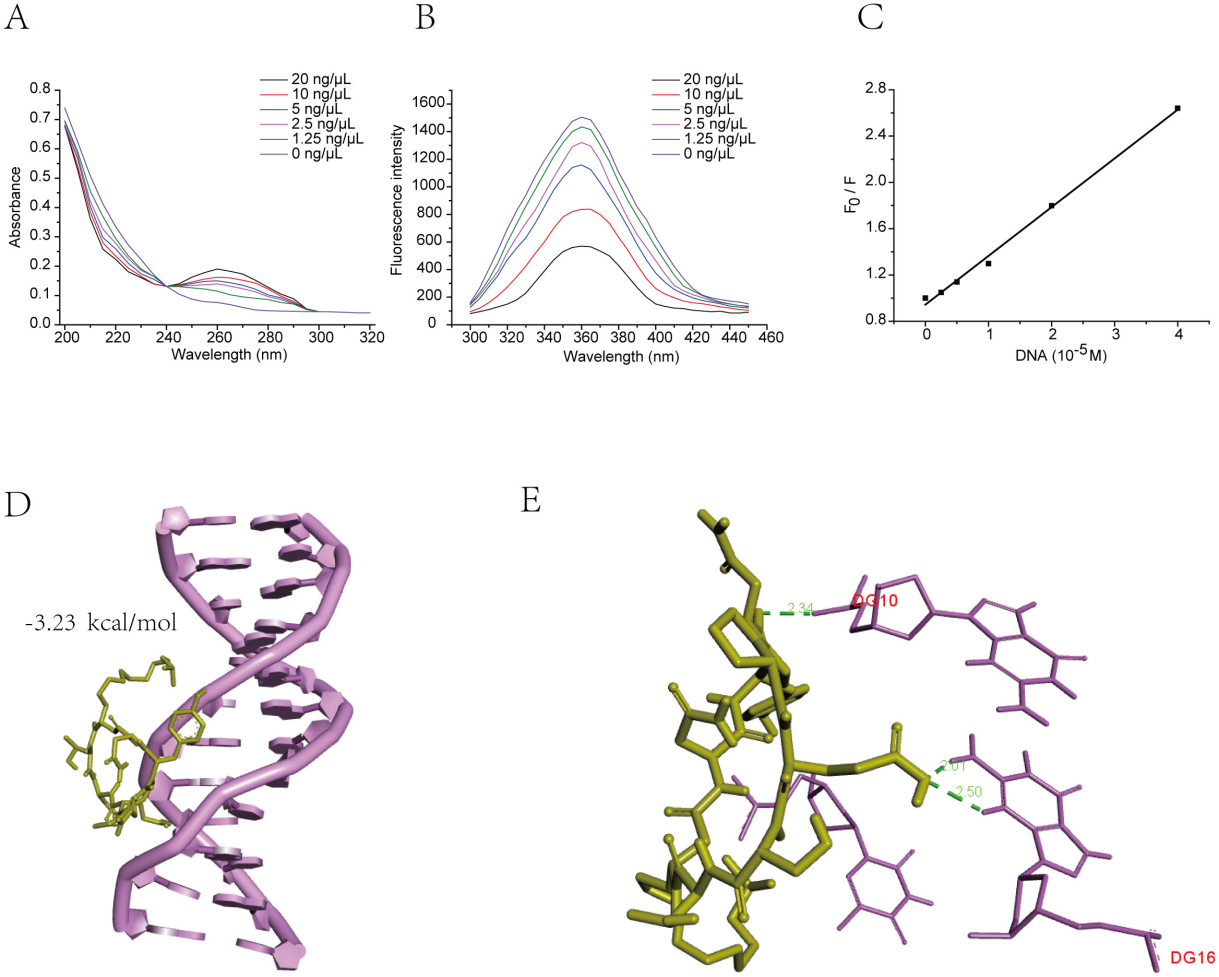
Effect of Bacillomycin D-C16 on DNA interaction. UV absorption spectra of Bacillomycin D-C16 during titration with increasing DNA concentrations **(A)**. Fluorescence intensity of Bacillomycin D-C16 with increasing concentrations of DNA **(B)**. The Stern–Volmer plots of Bacillomycin D-C16 by DNA **(C)**. Energy-minimized molecular docking models of Bacillomycin D-C16–DNA complexes **(D, E)**.

Figure 7A demonstrated the absorption spectra of Bacillomycin D-C16 upon interaction with varying DNA concentrations. As DNA was incrementally added, a mild decrease in Bacillomycin D-C16 absorption at 205 nm was observed without significant wavelength shift, accompanied by a distinct isosbestic point at 240 nm, demonstrating the formation of a stable complex between Bacillomycin D-C16 and DNA.

#### 3.5.2 Fluorescence spectroscopy

As demonstrated in Figure 7B, Bacillomycin D-C16 exhibited a prominent fluorescence emission peak at 360 nm. The fluorescence intensity significantly decreased upon DNA addition, indicating binding interactions between Bacillomycin D-C16 and DNA. The Stern-Volmer equation was applied to determine the binding constant (K_q_) as follows: F_0_/F = 1 + K_q_τ_0_[DNA] = 1 + K_sv_[DNA], where F_0_ and F represent the fluorescence intensities of free and DNA-bound Bacillomycin D-C16, respectively; [DNA] denotes genomic DNA concentration (mol L^−1^), τ_0_ is the average fluorescence lifetime of the molecule (10^−8^ s); and K_q_ is the bimolecular quenching rate constant. The value of K_q_ (3.16 ± 0.80 × 10^12^ L mol^−1^ s^−1^) was determined from the slope-to-intercept ratio of the linear regression. The linear regression analysis (*R*^2^ = 0.996) confirmed the bimolecular quenching mechanism (Figure 7C).

#### 3.5.3 Molecular modeling of Bacillomycin D-C16–DNA interaction

To investigate Bacillomycin D-C16 interactions with DNA, molecular docking simulations were performed. The ligand (Bacillomycin D-C16) was docked with DNA (Figures 7D, E). The simulations revealed that Bacillomycin D-C16 binds to the DNA major groove via hydrogen bonding, with a binding energy of −3.23 kcal/mol. Hydrogen bonds were formed between DG10, DG16, and Bacillomycin D-C16. The results indicate that Bacillomycin D-C16 interacts with DNA, where hydrogen bonding likely facilitates the binding mechanism.

## 4 Discussion

In this study, the MIC of Bacillomycin D-C16 (a Bacillomycin D monomer) against *F. oxysporum* was 8 mg/L, which was lower than the MIC values reported for fengycin B-C17 (250 mg/L) and iturin A-C14 (150 mg/L) (Deng et al., 2024; Wang et al., 2022). TEM analysis revealed that fungal cells exposed to Bacillomycin D-C16 exhibited distorted morphology, including: dissolution of the cell wall and membrane, reduction or dissolution of cytoplasmic organelles, extensive vacuolization, and complete mitochondrial disruption. The results indicate that Bacillomycin D-C16 exhibits strong inhibitory effects against *F. oxysporum*. As a lipopeptide surfactant, Bacillomycin D primarily compromises fungal cell membrane integrity (Liu et al., 2025), triggering secondary effects such as ROS accumulation, ATP synthesis inhibition, and TCA cycle perturbation (Lin et al., 2022; Wang S. et al., 2024). Beyond this canonical surfactant-mediated mechanism, we further employed transcriptomics and DNA-targeted molecular docking simulations to investigate potential complementary or novel antifungal pathways.

Transcriptomics technology plays a critical role in elucidating the molecular mechanisms of fungal inactivation by enabling quantitative and qualitative analyses of differential gene expression profiles (Chen et al., 2022; Yang et al., 2025). In the present study, we utilized RNA-Seq to analyze the transcriptional profile of *F. oxysporum* exposed to Bacillomycin D-C16. The results demonstrated significant alterations in gene expression following treatment, with DEGs predominantly associated with oxidative phosphorylation, TCA cycle, DNA replication, and glutathione metabolism pathways compared to the control group.

Oxidative phosphorylation and the TCA cycle, which take place in the mitochondria, are primary energy-producing pathways in eukaryotic cells (Martínez-Reyes and Chandel, 2020). Oxidative phosphorylation depends on mitochondrial complexes I–V to complete energy conversion, whereas the tricarboxylic acid (TCA) cycle supplies substrates (e.g., NADH and FADH_2_) required for oxidative phosphorylation (OuYang et al., 2018; Zhang C. et al., 2024). A recent study demonstrated that the antifungal activity of the antimicrobial peptide Epinecidin-1 against *Botrytis cinerea* results from severe disruption of oxidative phosphorylation and the TCA cycle (Fan et al., 2024). In the current study, transcriptome data showed that Bacillomycin D-C16 significantly down-regulated the expression of genes involved in oxidative phosphorylation pathway (NDUF, SDH, QCR, COX, and ATPase) and the TCA cycle (MDH, SDH, CS, IDH, ACN, SUC, FUM, PC, PDH, and LSC). To confirm this finding, we measured the enzymatic activities of mitochondrial complexes I-V along with MDH, IDH, and PDH. Exposure to Bacillomycin D-C16 significantly inhibited activities of both mitochondrial complexes I-V and the TCA cycle enzymes MDH, IDH, and PDH (*P* < 0.05; Figures 5F–J). Mitochondrial oxidative phosphorylation serves as the primary ATP-producing mechanism in cells under aerobic conditions, playing a critical role in maintaining intracellular ATP homeostasis. Inhibition of oxidative phosphorylation significantly reduces cellular ATP concentrations (Wang et al., 2015). Previous studies have demonstrated that mitochondrial electron transport chain inhibitors diminish mitochondrial membrane potential (MMP) by suppressing proton translocation across the respiratory chain complexes, ultimately decreasing ATP synthesis and inducing cell death (Kaim and Dimroth, 1999; OuYang et al., 2018). In this study, we observed that Bacillomycin D-C16 significantly reduced ATP levels and diminished MMP (Figures 5A, B). This result aligns with the findings of Wang S. et al. (2024), which demonstrated that treatment with the lipopeptide Iturin A induces mitochondrial morphological changes, suppresses ATP production, and inhibits the TCA cycle in *Aspergillus niger*. The results demonstrate that Bacillomycin D-C16 treatment disrupts both oxidative phosphorylation and the TCA cycle, leading to collapse of MMP, impaired ATP synthesis, and generalized mitochondrial dysfunction.

Transcriptomic analyses consistently demonstrated that Bacillomycin D-C16-induced *F. oxysporum* cell death was associated with glutathione metabolism. This pathway involves glutathione S-transferases (GSTs), which catalyze glutathione (GSH) conjugation with xenobiotic compounds (Kong et al., 2024). Specifically, we observed 2.84-fold and 2.48-fold upregulation of two GST-encoding genes (FOYG_15816 and FOYG_1936), consistent with enhanced GST enzymatic activity that directly reduced intracellular GSH levels (Figures 6B, C). Since GSH is essential for detoxifying ROS and maintaining redox homeostasis, its depletion likely induced sustained ROS accumulation, thereby impairing fungal viability through oxidative damage (Qin et al., 2023; Xin et al., 2024). This hypothesis was further supported by significantly higher fluorescence values observed exclusively in samples treated with 1 MIC Bacillomycin D-C16 (Figure 6A). These findings align with reports on other antifungal agents. OuYang et al. (2018) demonstrated that citral stimulated GST activity in *Penicillium digitatum* while geranial treatment reduced intracellular GSH levels, concomitant with increased ROS. Similarly, antifungal peptides PPD1-FRLHF, 66-10-FRLKFH, and 77-3-FRLKFHF were shown to decrease reduced glutathione content and induce ROS bursts in *Aspergillus flavus*, contributing to cell death (Devi et al., 2021). The antioxidants VC, GSH, and NAC effectively neutralized cellular ROS and significantly attenuated Bacillomycin D-C6′s fungicidal activity (Figure 6E). This protective effect is consistent with the findings of Liu et al. (2019b) on fengycin A-C6′s antifungal activity against *Candida albicans*, and further confirms that ROS accumulation enhances Bacillomycin D-C16-induced cellular damage in *Fusarium oxysporum*. Collectively, these results establish ROS accumulation as a key mechanism underlying Bacillomycin D-C6′s fungistatic effects.

Antimicrobial peptides with diverse modes of action can penetrate cell membranes and engage intracellular targets like DNA (Fan et al., 2023, 2024; Liu et al., 2019a). DNA replication is the foundation of biological inheritance, ensuring the accurate transmission and preservation of genetic information. Aberrant DNA replication can lead to genetic mutations or even cell death (Putnam, 2021). In the current study, nearly all DEGs associated with DNA replication exhibited significant down-regulation. The proteins encoded by these DEGs are critical to multiple stages of DNA replication. For instance, DNA helicases encoded by MCM4 unwind double-stranded DNA during replication initiation (Bell and Dutta, 2002). The down-regulation of such genes suggests inhibition of the replication initiation phase. Furthermore, genes associated with DNA replication elongation, including those encoding replication factors (RFC1, RFC2), DNA ligase (Lig1), the DNA polymerase subunit (POLD1), and DNA primase (PRI2), were also markedly downregulated. The reduced expression of these genes indicates suppression of the DNA replication. Notably, certain antifungal agents exhibit DNA-intercalating properties, which can disrupt replication machinery to exert antifungal effects (Wang et al., 2024; Zhang et al., 2017). UV absorption and fluorescence spectroscopy confirmed Bacillomycin D-C16′s binding to the grooves of DNA. Molecular docking simulations further corroborated these findings by illustrating its preferential binding within the major DNA groove, consistent with the interaction mechanisms reported by Liu et al. (2019a). Collectively, these results indicate that Bacillomycin D-C16 induces *F. oxysporum* cell death by inhibiting DNA replication through groove-binding-mediated mechanisms, as evidenced by transcriptomic and molecular simulation studies.

## 5 Conclusion

**Figure 8:**
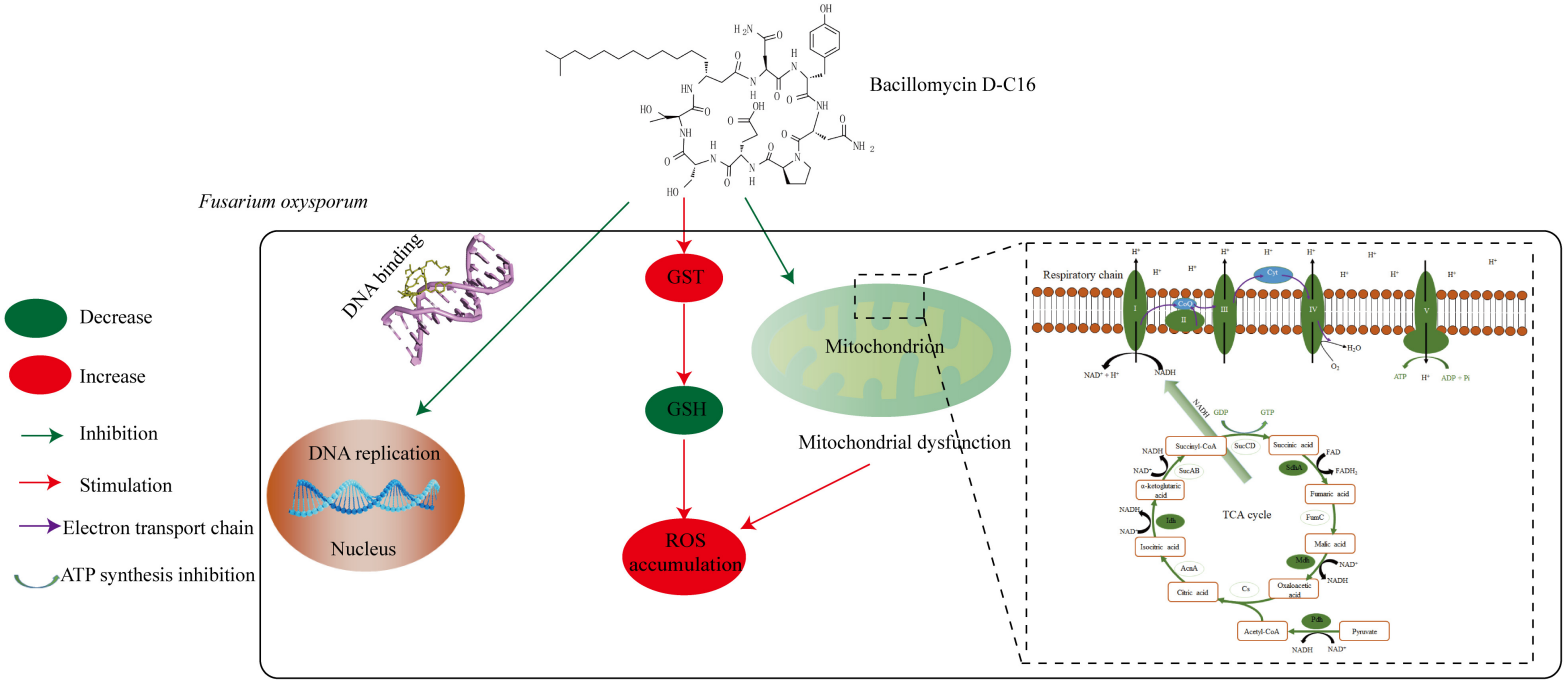
The potential antifungal mechanism of Bacillomycin D-C16 against *F. oxysporum*.

This study demonstrated that Bacillomycin D-C16 exhibited significant antifungal activity against *F. oxysporum*. The compound primarily induced mitochondrial membrane damage, disrupting both mitochondrial structure and function. Consequently, it inhibited oxidative phosphorylation and the TCA cycle, thereby blocking energy metabolism. Additionally, Bacillomycin D-C16 decreased GSH content while promoting reactive oxygen species (ROS) accumulation. Notably, the lipopeptide also displayed DNA-binding capability and suppressed DNA replication. These findings suggest that Bacillomycin D-C16 possesses substantial potential for fungicide development. The potential antifungal mechanism of Bacillomycin D-C16 against F. oxysporum is illustrated in Figure 8.

## Data Availability

The data presented in this study are publicly available. The data can be found here: https://www.ncbi.nlm.nih.gov/bioproject, accession PRJNA1290884.
