# Supplementary material for: Antifungal mechanism and transcriptome analysis of Bacillomycin D-C16 against Fusarium oxysporum
Source: Front Microbiol. 2025 Nov 25;16:1698200. doi: 10.3389/fmicb.2025.1698200 (PMC12687139; doi:10.3389/fmicb.2025.1698200)
Supplement: Supplementary file 1 [file Table_1.docx]

Table S1 Primer sequence for RT-PCR

| Gene function annotation | Gene ID | Revers primer (5'→3') |
| --- | --- | --- |
| *NDH* | FOYG_02742 | F: AAGTTCGAGCAGGCTTCTCT |
|  |  | R: TTGGCCAAGTCCTCGTGTAT |
| *ATPase* | FOYG_16236 | F: CGAAGATCAGCTCCAAACCG |
|  |  | R: CGAACTGAATTGGGCCTACG |
| *Cyt1* | FOYG_06852 | F: TCTCGTTGGTTCCGTCTTGA |
|  |  | R: GGAAGCATGTAATCGGCCAG |
| *CYC* | FOYG_10823 | F: TCTTCAAGACCCGATGTGCT |
|  |  | R: CTTCTTGGGGTTCTCGAGGT |
| *MDH* | FOYG_08718 | F: AGACAACTGGCTACCTTCCC |
|  |  | R: TCGTCACGAGTCATACCAGG |
| *IDH* | FOYG_05608 | F: ACAGGAGCTCGACATCTACG |
|  |  | R: CTCGGTGTTCTCTCGGATGA |
| *GST* | FOYG_01936 | F: TGCAGGAGAGGAGTTTACGG |
|  |  | R: GTCTTCTTGAAGCCCTCCCT |
| *EF1-α* |  | F: AGCTCGGTAAGGGTTCCTTC |
|  |  | R: TCCAGAGAGCAATATCGATGG |
