# Supplementary material for: Antifungal mechanism and transcriptome analysis of Bacillomycin D-C16 against Fusarium oxysporum
Source: Front Microbiol. 2025 Nov 25;16:1698200. doi: 10.3389/fmicb.2025.1698200 (PMC12687139; doi:10.3389/fmicb.2025.1698200)
Supplement: Supplementary file 2 [file Table_2.docx]

Table S2 Overview of the transcriptome sequencing dataset and quality check

| Samples | Raw reads | Clean reads | Q20(%) | Q30(%) | GC content(%) |
| --- | --- | --- | --- | --- | --- |
| CK_1 | 44055052 | 43620562 | 97.94 | 93.88 | 52.29 |
| CK_2 | 41026826 | 40618524 | 97.77 | 93.38 | 52.35 |
| CK_3 | 46485740 | 46095742 | 98.01 | 94.06 | 52.36 |
| BD_1 | 46335862 | 45884168 | 97.78 | 93.45 | 51.97 |
| BD_2 | 50178058 | 49721502 | 97.88 | 93.72 | 51.98 |
| BD_3 | 51950932 | 51496994 | 97.92 | 93.82 | 51.91 |

Note: BD, Bacillomycin D-C16. CK, control.
