# Supplementary figures and images for: Antifungal mechanism and transcriptome analysis of Bacillomycin D-C16 against Fusarium oxysporum
Source: Front Microbiol. 2025 Nov 25;16:1698200. doi: 10.3389/fmicb.2025.1698200 (PMC12687139; doi:10.3389/fmicb.2025.1698200)

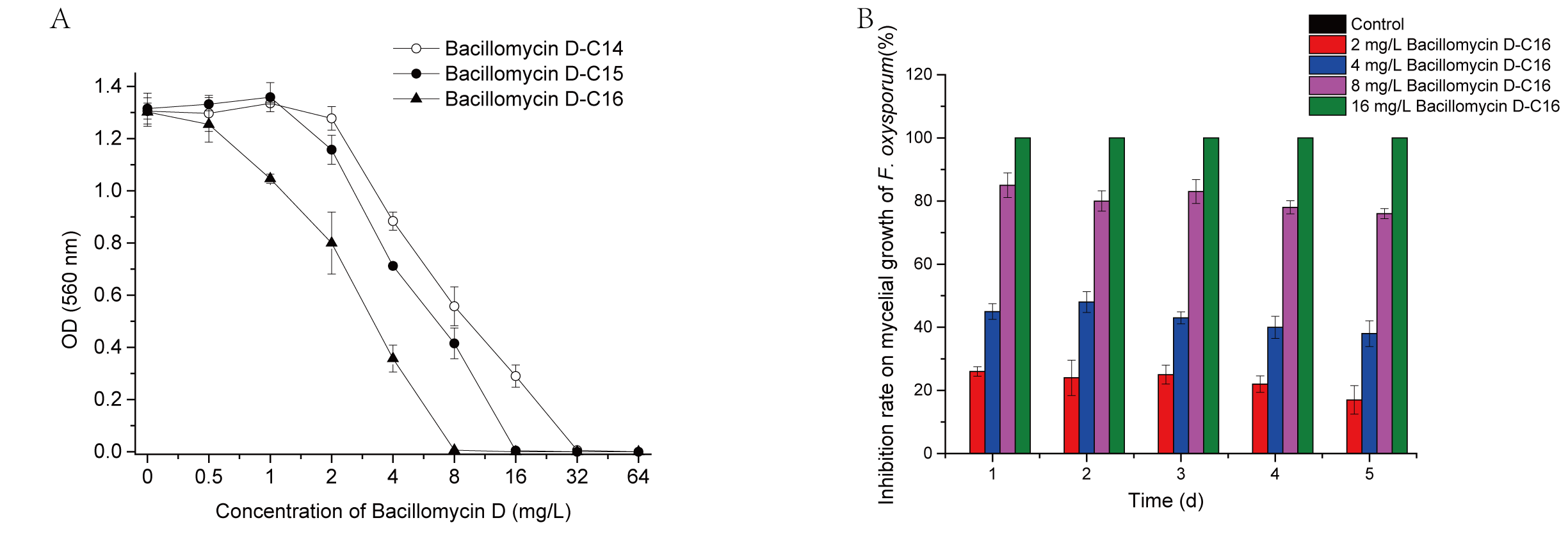

Supplement: Supplementary file 8 [file Image_1.tif]

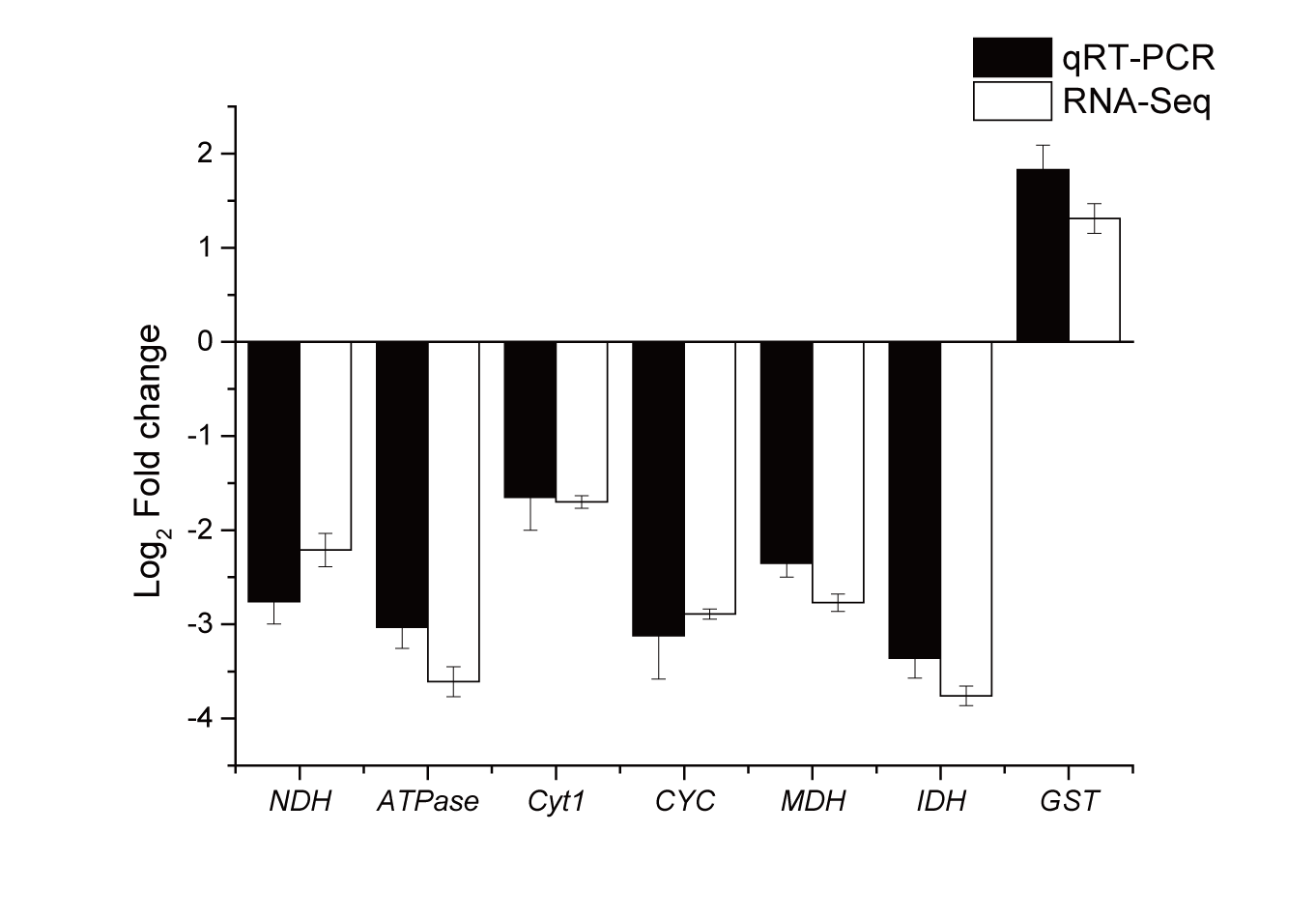

Supplement: Supplementary file 9 [file Image_2.tif]
